# Meta-Analysis of Gene Expressions in Testicular Germ Cell Tumor Histologies

**DOI:** 10.3390/ijms21124487

**Published:** 2020-06-24

**Authors:** Finn Edler von Eyben, Jorge Parraga-Alava

**Affiliations:** 1 Center of Tobacco Control Research, DK-5230 Odense M, Denmark; 2 Facultad de Ciencias Informaticas, Universidad Tecnica de Manabi, Portoviejo 130105, Ecuador; jorge.parraga@usach.cl; 3 Departamento de Ingenieria Informatica, Universidad de Santiago de Chile, Santiago 9170020, Chile

**Keywords:** biomarkers, genes for pluripotency, meta-analysis, oncogenes, pathogenesis, RNA expression, testicular neoplasms, tumor suppressor genes

## Abstract

There is no consensus as to how a precursor lesion, germ cell neoplasia in situ (GCNIS), develops into the histologic types of testicular germ cell tumor type II (TGCT). The present meta-analysis examined RNA expressions of 24 candidate genes in three datasets. They included 203 samples of normal testis (NT) and histologic types of TGCT. The Fisher’s test for combined *p* values was used for meta-analysis of the RNA expressions in the three datasets. The histologic types differed in RNA expression of *PRAME, KIT, SOX17, NANOG, KLF4, POU5F1, RB1, DNMT3B*, and *LIN28A* (*p* < 0.01). The histologic types had concordant differences in RNA expression of the genes in the three datasets. Eight genes had overlap with a high RNA expression in at least two histologic types. In contrast, only seminoma (SE) had a high RNA expression of *KLF4* and only embryonal carcinoma (EC) had a high RNA expression of *DNMT3B*. In conclusion, the meta-analysis showed that the development of the histologic types of TGCT was driven by changes in RNA expression of candidate genes. According to the RNA expressions of the ten genes, TGCT develops from NT over GCNIS, SE, EC, to the differentiated types of TGCT.

## 1. Introduction

Testicular germ cell tumor type II (TGCT) is the most frequent malignancy in young adult men. Worldwide, oncologists follow the TNM (T for primary tumor, N for regional lymph node metastases, M for distant metastases) classification that separates TGCT into seminoma and nonseminomatous germ cell tumors (NSGCT) [1].

The WHO classification of tumors of the urogenital system and male genital organs 2016 [2] acknowledges that TGCT develops from a common precursor lesion, germ cell neoplasia in situ (GCNIS). The WHO classification is based on extensive previous research. The research started in 1896, in which Wilms reported that TGCT was derived from normal testicular germ cells [3].

In 1950, Dixon and Moore described that TGCT developed along two lines, i.e., one line for seminoma and the other line for NSCGT [4]. With regards to NSGCT, embryonal carcinoma (EC) could differentiate into teratoma (TER) and choriocarcinoma (CC). In 1959, Teilum described a separate histologic type of NSGCT, YST (also called endodermal sinus tumor) [5]. In 1990, de Jong summarized studies that showed TGCT had a characteristic isochromosome of the short arm of chromosome 12, 12p, and i(12p) [6].

In 2004, in a meta-analysis of genes in TGCT, von Eyben pointed out that undifferentiated types of TGCT had high levels of CCND2 and low levels of RB1 which combined, programed the malignant germ cells for a high proliferation [7]. A meta-analysis by Alagaratnam, in 2011, pointed out that 92 genes had roles for the pathogenesis of TGCT [8]. From 2012 to 2016, Nettersheim in studies of xenografts of TCam2 to nude mice showed that seminoma (SE) can develop into EC [9].

After the WHO classification in 2016, an immunohistochemical study, in 2017, showed that precursor lesions for TGCT had a seminomatous immunophenotype [10]. The Genome Cancer Atlas (TGCA) project, in 2018, found that TGCT did not have mandatory somatic mutations [11].

In general, solid tumors develop as a result of an accumulation of mutations or epigenetic changes in oncogenes or tumor suppressor gene. As TGCT does not have mandatory mutations, the development of the histologic types should be caused by regulation of gene expression of candidate genes. To further study how TGCT develops, we aimed to investigate whether the histologic types of TGCT differed significantly in RNA expression of candidate genes.

## 2. Results

### 2.1. Comparisons of Three Datasets

Our meta-analysis examined RNA expression of 24 candidate genes in three datasets of TGCT. The three datasets are publicly available [12,13,14]. Previous publications had reported that the gene products of the 24 candidate genes were significant in the pathogenesis or treatment of TGCT.

The three datasets were from studies that differed with respect to time, country, and methodology for the RNA measurements. Nevertheless, the histologic types had concordant RNA expressions of eight significant genes. Therefore, our meta-analysis combined the evaluation of the three datasets.

We combined *p* values for overlap of RNA expressions of concordant genes in the histologic types from the *p* values of the individual three RNA datasets, as shown in Table 1 [15]. The histologic types also differed in RNA expressions of genes, as shown in Table 2.

### 2.2. Significant Genes

**Figure 1:**
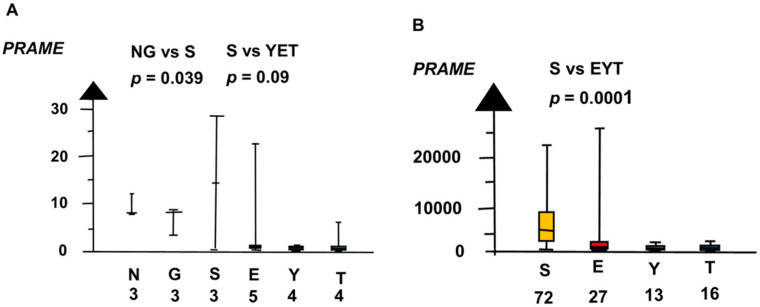
RNA expression of *PRAME* in the two microarrays (**A**,**B**) show that normal testis (N), germ cell neoplasia in situ (G) and seminoma (S) had high RNA expression and embryonal carcinoma (E), yolk sac tumor (Y), and teratoma (T) had a low RNA expression. The boxes show the median and the upper and lower quartiles and the whiskers show the full range.

Our meta-analysis found the histologic types had overlaps and differences in RNA expressions of the candidate genes. Normal testis (NT), GCNIS, and SE had overlap with high RNA expression of *PRAME*, as shown in Figure 1. In the second microarray, NT, GCNIS, and SE combined had a sixteen times higher RNA expression of *PRAME* than EC, YST, and TER combined (median 8.4 vs. 0.4, *p* = 0.003, Kruskal–Wallis test).

**Figure 2:**
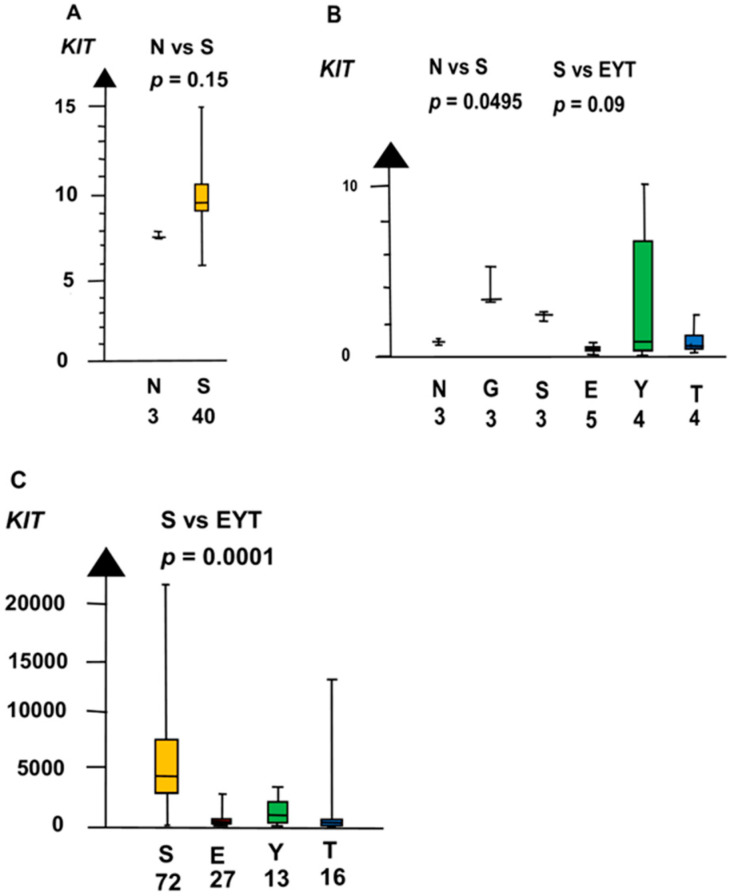
RNA expression of *KIT* in the three datasets (**A**–**C**) show that germ cell neoplasia in situ (G) and seminoma (S) had high RNA expression and that embryonal carcinoma (E), yolk sac tumor (Y), and teratoma (T) had a low RNA expression.

GCNIS had a higher RNA expression than NT of three genes, i.e., *KIT, SOX17*, and *NANOG*. In the second microarray, GCNIS and SE combined had a four times higher RNA expression of *KIT* than NT (median 4.3 vs. 0.89, *p* = 0.02, Kruskal–Wallis test), as shown in Figure 2.

**Figure 3:**
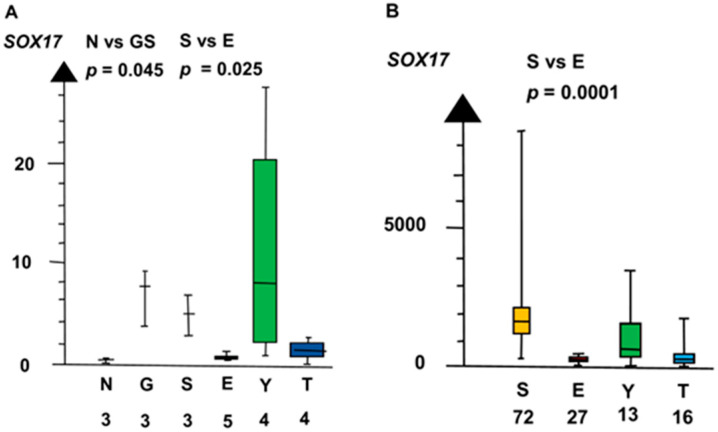
RNA expression of *SOX17* in the two microarrays (**A**,**B**) show that germ cell neoplasia in situ (G), seminoma (S), and yolk sac tumor (Y) had a high RNA expression and that embryonal carcinoma (E) and teratoma (T) had a low RNA expression.

GCNIS and SE combined had a four times higher RNA expression of *SOX17* than EC, YST, and TER combined (median 3.9 vs. 1.0, *p* = 0.023, Kruskal–Wallis test), as shown in Figure 3.

**Figure 4:**
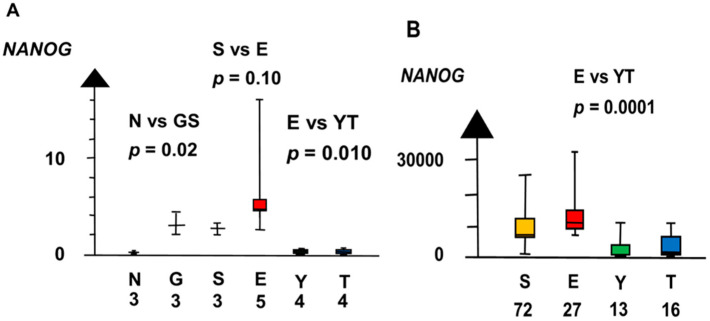
RNA expression of *NANOG* in the second microarray and the RNAseq dataset (**A**,**B**) show that germ cell neoplasia in situ (G), seminoma (S), and embryonal carcinoma (E) had a high RNA expression and that yolk sac tumor (Y) and teratoma (T) had a low RNA expression.

GCNIS and SE had a ten times higher RNA expression of *NANOG* than NT (median 2.9 vs. 0.29, *p* = 0.02, Kruskal–Wallis test), as shown in Figure 4. *NANOG* differed stepwise among histologic types. SE and EC combined had a three times higher RNA expression of *NANOG* than NT and GCNIS combined (median 3.8 vs. 1.2, *p* = 0.039, Kruskal–Wallis test).

SE had a set of overexpressed genes that differed from that in GCNIS. The RNA expression of *CCND2, KLF4*, and *POU5F1* was high in SE but low in GCNIS. Furthermore, SE had a downregulated tumor suppressor gene, *RB1*, not downregulated in GCNIS. In contrast, both GCNIS and SE had high RNA expression of *KIT, SOX17*, and *NANOG*.

**Figure 5:**
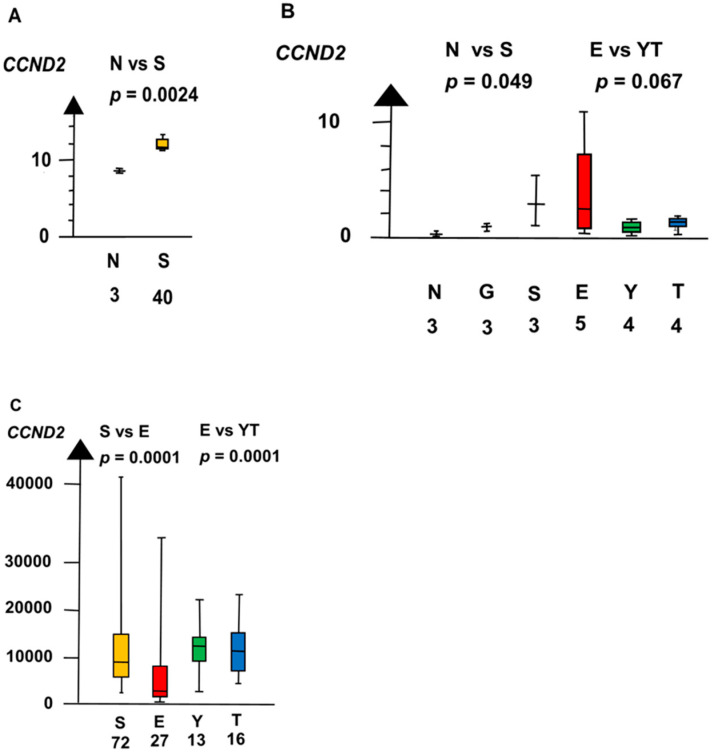
RNA expression of *CCND2* in the three datasets (**A**–**C**) show that germ cell neoplasia in situ (G), seminoma (S), and embryonal carcinoma (E) had a high RNA expression and that yolk sac tumor (Y) and teratoma (T) had a low RNA expression.

In the second microarray, SE had a twelve times higher RNA expression of *CCND2* than NT (median 3.1 vs. 0.26, *p* = 0.0495, Kruskal–Wallis test), as shown in Figure 5.

**Figure 6:**
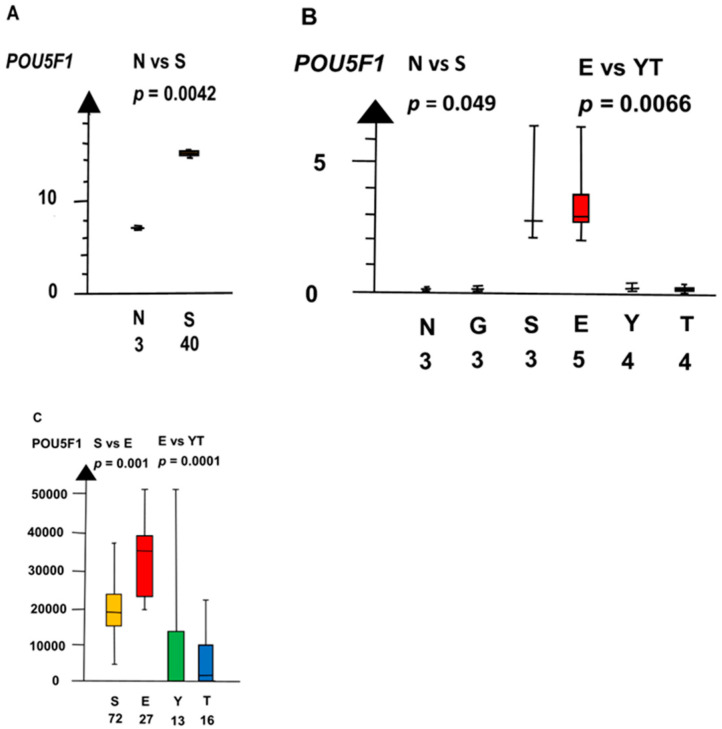
RNA expression of *POU5F1* in three datasets (**A**–**C**) show that seminoma (S) and embryonal carcinoma (E) had a high RNA expression and that germ cell neoplasia in situ (G), yolk sac tumor (Y), and teratoma (T) had a low RNA expression.

SE and EC combined had a four times higher RNA expression of *POU5F1* than NT and GCNIS combined (median 3.5 vs. 0.72, *p* = 0.0045, Kruskal–Wallis test), as shown in Figure 6.

**Figure 7:**
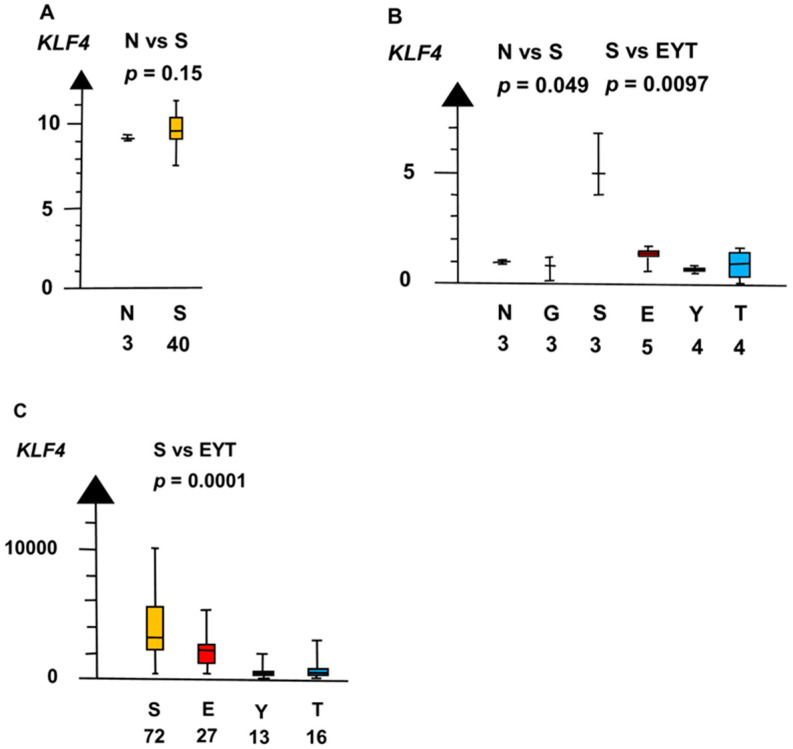
RNA expression of *KLF4* in the three datasets (**A**–**C**) show that seminoma (S) had a high RNA expression and that germ cell neoplasia in situ (G), embryonal carcinoma (E), yolk sac tumor (Y), and teratoma (T) had a low RNA expression.

SE and EC had a seven times higher RNA expression of *KLF4* than all other histologic types combined (median 4.9 vs. 0.71, *p* = 0.0073, Kruskal–Wallis test), as indicated in Figure 7.

**Figure 8:**
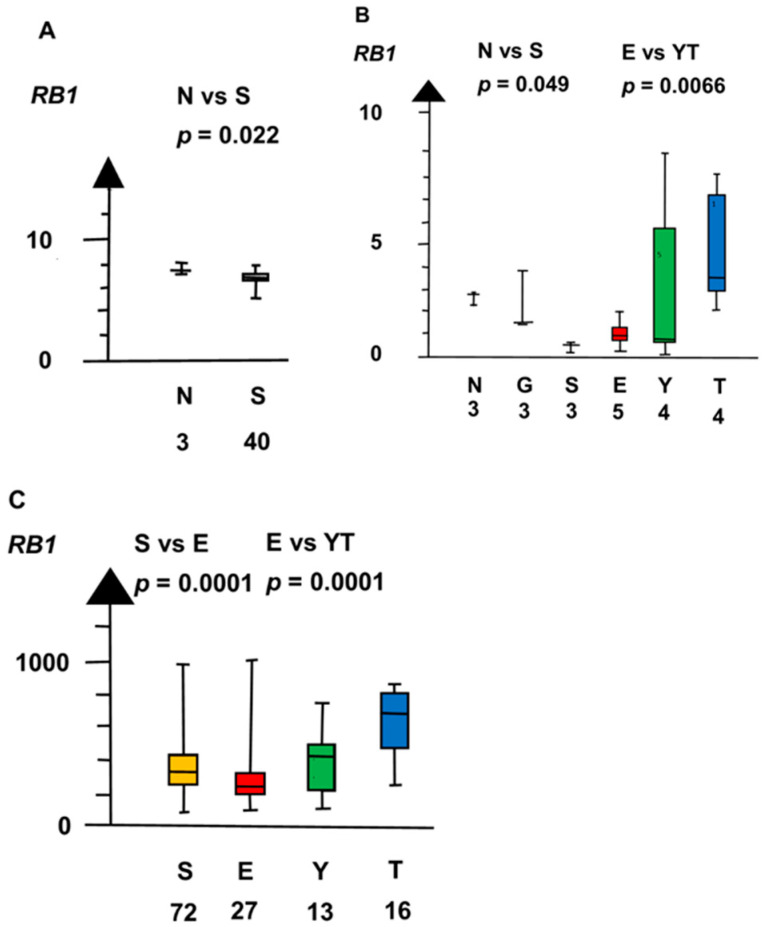
RNA expression of *RB1* in the three datasets (**A**–**C**) show that seminoma (S) and embryonal carcinoma (E) had a low RNA expression and that teratoma (T) had a high RNA expression.

SE and EC combined had an RNA expression of *RB1* that was a quarter of that of NT and GCNIS combined (median 0.5 vs. 2.3, *p* = 0.0495, Kruskal–Wallis test), as shown in Figure 8.

EC had a set of genes with overexpressed RNA that differed from that in SE. EC had high RNA expressions of *DMT3B* and *LIN28A*, but the RNA expressions were low in SE.

**Figure 9:**
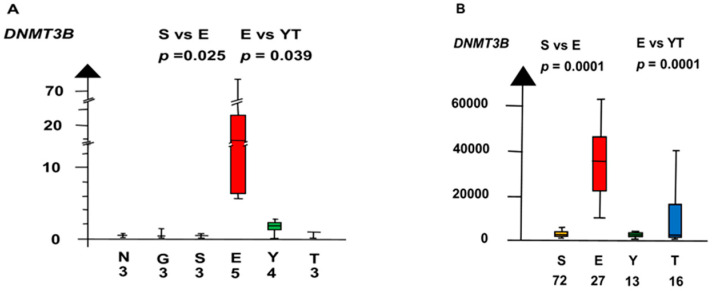
RNA expression of *DNMT3B*. in the second microarray and the RNAseq dataset (**A**,**B**) show that embryonal carcinoma (E) had a high RNA expression and that germ cell neoplasia in situ (G), seminoma (S), yolk sac tumor (Y), and teratoma (T) had a low RNA expression.

In the second microarray, EC had an 18 times higher RNA expression of *DNMT3B* than all other histologic types combined (median 18.3 vs. 1, *p* = 0.0013, Kruskal–Wallis test), as shown in Figure 9.

**Figure 10:**
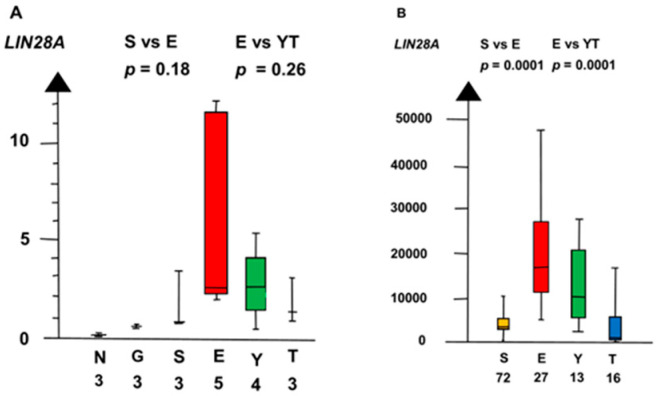
RNA expression of *LIN28A* in the second microarray and the RNAseq dataset (**A**,**B**) show that embryonal carcinoma (E) had a high RNA expression and that yolk sac tumor (Y), and teratoma (T) had a low RNA expression.

EC had a three times higher RNA expression of *LIN28A* than SE (2.6 vs. 0.85, p = 0.18, Kruskal–Wallis test), as shown in Figure 10. *LIN28A* differed stepwise among the histologic types. SE and EC combined had a six times higher RNA expression of *LIN28A* than NT and GCNIS combined (median 2.4 vs. 0.36, *p* = 0.0019, Kruskal–Wallis test).

Differentiated NSGCT, TER, YST, and CC, had genes that were overexpressed, whereas EC did not overexpress the genes. TER had a nine times higher RNA expression of *RB1* than EC (median 4.6 vs. 0.5, *p* = 0.028, Kruskal–Wallis test), as shown in Figure 8. YST had a 21 times higher RNA expression of *AFP* than EC (median 9.4 vs. 0.4, *p* = 0.02, Kruskal–Wallis test).

CC had an eleven times higher RNA expression of *CGB5* than EC (9.0 vs. median 0.8, *p* = 0.14, Kruskal–Wallis test).

### 2.3. Nonsignificant Genes

In the second microarray, the histologic types did not differ significantly in RNA expression of *CDKN1A, CDKN2C. CCNE1, FOXD0, MYC, MYCN, MDM2*, and *PTTG1*.

### 2.4. Role of Genes for the Development of Testicular Germ Cell Tumor Type II (TGCT)

**Figure 11:**
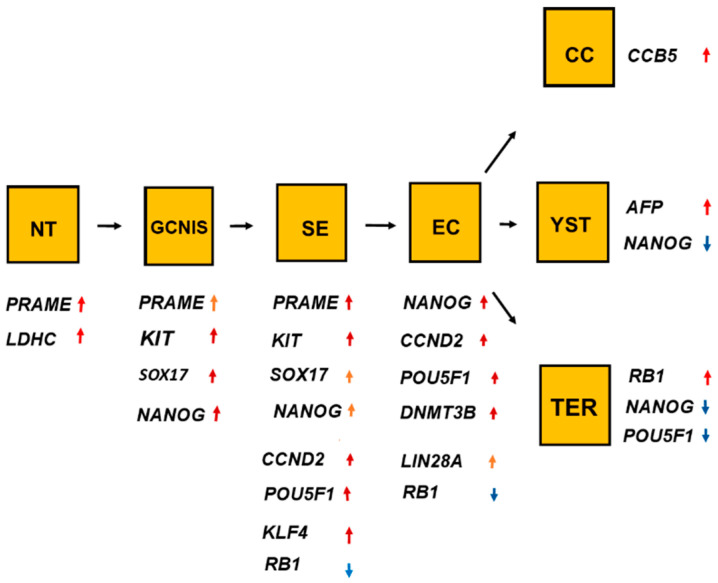
The overlaps and differences of the ten genes shown in Figure 1, Figure 2, Figure 3, Figure 4, Figure 5, Figure 6, Figure 7, Figure 8, Figure 9 and Figure 10 showed that one gene was upregulated in normal testis (NT), and other genes were upregulated in germ cell neoplasia in situ (GCNIS). Undifferentiated histologic types, seminoma (SE) and embryonal carcinoma (EC), differed in RNA expression. EC differed in RNA expression from the differentiated histologic types such as choriocarcinoma (CC), yolk sac tumor (YST), and teratoma (TER). The figure indicates the linkage among the histologic types. The change from GNIS to SE was associated with a change in RNA expression of three significant genes. As for the genes, red arrows denote increased RNA expression and the blue arrows denote reduced RNA expression.

The histologic types of TGCT had considerable overlaps of high RNA expressions of many genes, as shown in Table 1 and Figure 11. The overlaps indicate the histologic types have close relations during the development.

The histologic types also differed in RNA expression of significant genes, as shown in Table 2 and Figure 11. The following two genes were genetic signatures for a histologic type: *KLF4* for SE and *DNMT3B* for EC. The significant differences for RNA expressions of genes among histologic types indicate that the histologic types had different roles for the genes.

**Figure 12:**
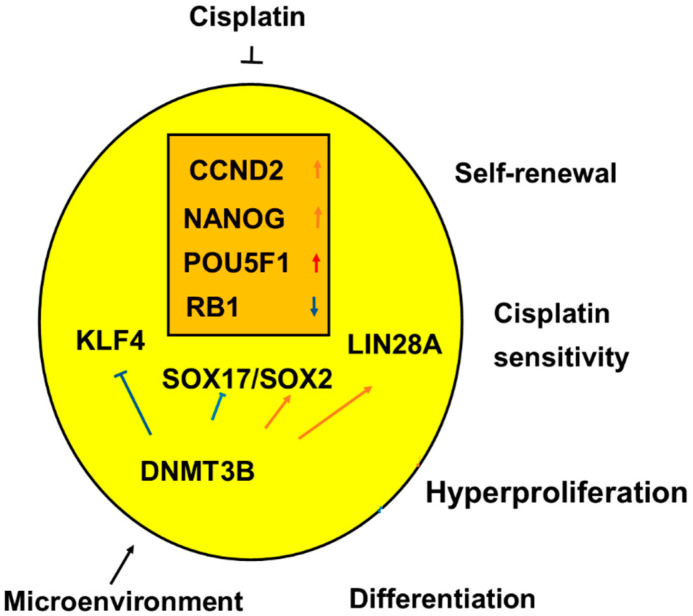
The undifferentiated histologic types of testicular germ cell tumor type II (TGCT), seminoma (SE), and embryonal carcinoma (EC) had overlap with upregulation of three genes shown with red arrows and downregulation of one gene shown with a blue arrow in the orange box. Microenvironmental factors can upregulate *DNMT3B* that blocks *SOX17* and *KLF4* and upregulates *SOX2* and *LIN28A*, genetic changes associated with the histologic transition from a seminomatous cell type to an embryonal carcinoma cell type. Significant genes are associated with the main features of the malignant undifferentiated germ cells such as self-renewal, hyperproliferation, and sensitivity to platin-based chemotherapy. As for the genes, red arrows denote increased RNA expressions or a stimulus of genes and the blue arrows denote reduced RNA expression or an inhibition of genes.

In undifferentiated histologic types of TGCT, SE, and EC, high RNA expressions of significant genes are associated with the main clinical features such as self-renewal, hyperproliferation, and sensitivity to platin-based chemotherapy, as indicated in Figure 12.

## 3. Discussion

Our meta-analysis of cancer genomics of 24 candidate genes of TGCT fulfilled the major goal to identify driver genes for initiation of GCNIS, the precursor lesion, and progression to the histologic types of TGCT. The histologic types differed significantly in RNA expression of *PRAME, KIT, SOX17, NANOG, CCND2, KLF4, POU5F1, RB1, DNMT3B*, and *LIN28A*.

Eight of the ten significant candidate genes differed concordantly in the three datasets regarding RNA expressions among the histologic types. Thus, our gene analyses indisputably support that NT develops over GCNIS to SE, EC, and the differentiated types of NSGCT.

Our meta-analysis evaluated the individual tissue samples in the three datasets for the RNA expressions. Combined in a meta-analysis of the *p* values of the three datasets by the Fisher’s test [15], the summary *p* findings were highly significant.

Undifferentiated types of TGCT had a high RNA expression of *CCND2* and *NANOG* and both genes have their gene locus on chromosome 12p (12p13.32 and 12p13.31, respectively). Thus, the RNA expressions of the two genes in part explains how the high copy numbers of chromosome 12p in TGCT contribute to the pathogenesis of TGCT.

Although the recent WHO classification 2016 gave the precursor lesion an ambiguous name, GCNIS, the precursor lesion in reality is a localized malignancy. Half the GCNIS progress to microinvasive germ cell tumors within five years [16]. GCNIS was associated with high RNA expression of three genes, and SE was associated with high expression of two other genes and a reduced expression of a tumor suppressor gene.

SE can progress to EC. Both SE and EC, had high RNA expression of *CCND2, NANOG*, and *POU5F1* and, furthermore, both SE and EC had reduced RNA expression of *RB1*. The stepwise increase among GCNIS and the undifferentiated types of TGCT in levels of RNA expression of the three genes supports that the genes are driver genes in the pathogenesis of TGCT.

SE and EC also differed. SE had high RNA expressions of *PRAME, KIT*, and *SOX17*, not found in EC. EC had a high RNA expression of *LIN28A* not found in SE. SE had *KLF4* as a genetic signatures and EC had *DMNT3B* as a genetic signature. However, the differences between SE and EC do not prove that SE and EC develop according to separate lines.

The genes have two roles in the pathogenesis of TGCT. Overlap of high RNA expression of many genes in the histologic types bind the histologic types together. Differences in genes among the histologic types can be primary or secondary. Two genes had a high RNA expression only in one histologic type as a genetic signature. The two genes contribute to the specific characteristics of SE and EC.

For SE, *KLF4* is a genetic signature. *KLF4* could block SE for differentiation into differentiated types of NSGCT, TER, YST, and CC. RNA expression of *KLF4* had a longer half-life when the cells expressed NANOG and POU5F1 [17].

DNMT3B methylates DNA. For EC, *DNMT3B* is a genetic signature. The high RNA expression of *DNMT3B* causes EC to have more methylation of DNA than SE. Similarly, a proteomic study found that EC had high protein expression of DNMT3B [18]. Methylation of an upstream region for *POU5F1* regulated expression of *POU5F1* [18]. Downregulation of *DNMT3B* is part of the difference in gene expressions as EC differentiates into TER, YST, and CC.

Our results are in accordance with previous research. Undifferentiated and differentiated histologic types of TGCT differed in RNA expression of genes for pluripotency and, similarly, the two histologic types differed in protein levels of the genes in previous publications. Thus, TGCT mainly regulates the proteins at the step of transcription of the genes, like eukaryotic cells, in general, regulate the protein levels.

Our findings are consistent with an experimental study that xenografted a seminoma cell line, TCam2, to the flanks of nude mice, and therefore the TCam2 thereby developed into EC [19,20,21]. During the transition of TCam2 into EC, the bromodomain family of genes upregulated *DNMT3B* and *LIN28A* [20,21]. Both SE and EC had high RNA expression of *NANOG* and *POU5F1*.

The mechanisms caused EC, in our meta-analysis, to have similarly higher RNA expression of *DNMT3B, LIN28A, NANOG*, and *POU5F1* than SE, similar to the mechanisms reported in the experimental study.

In addition, a previous systematic review [7] showed that TGCT upregulated *CCND2* and downregulated *RB1*. Our findings were also consistent with the observations that GCNIS overexpressed genes for pluripotency [22], that MYC did not have a significant role in the development of the histologic types of TGCT [23], and that EC and the differentiated types of NSGCT differed in the level of the proteins for pluripotency [24].

Further previous studies also showed that SE and NSGCT had higher expression of *NANOG* and *POU5F1* than GCNIS, YST, and TER [25,26]. The differences between EC and differentiated NSGCT in our meta-analysis for *NANOG, POU5F1*, and *SOX17* are like the findings in another study as EC differentiates into TER. Methylations of CpG dinucleotides in NANOG regulatory regions downregulated gene expression of *NANOG* [27].

Importantly, our meta-analysis showed that genes for pluripotency were upregulated in SE and EC and not only in GCNIS. The genes had a stepwise increase in RNA expression from NT over GCNIS, and SE to EC and a reduction in TER. The changes indicate that epigenetics regulates the RNA expression of the genes.

Epigenetics for GCT can involve that methylations and acetylations of specific lysines on histone 3 (H3K2, H3K9, and H3K27) in promoters and enhancers of the significant candidate genes regulate the RNA expression of the genes [28,29,30].

NANOG can act together with SOX2 and POU5F1 and the complex of transcription factors can bind to a motif in DNA [31]. The complex of NANOG, POU5F1, and SOX2 especially increased RNA expression of *NANOG* in cells with a bivalent promoter for *NANOG*.

A panel of four genes for pluripotency including *KLF4, LIN28A, MYC, NANOG*, and *POU5F1* can reprogram somatic cells into induced pluripotent stem cells, iPSC [32,33]. The panel of genes for pluripotency is crucial in developmental biology. The genes for pluripotency can also be driver genes in the pathogenesis of TGCT type II in general.

As proof of the impact in patients with TGCT of genes for pluripotency, a set of genes for pluripotency could reprogram fibroblasts into iPSC in a report of a seminoma patient [34].

Both EC and embryonal stem cells (ESC) have a high expression of genes for pluripotency [35], but iPSC has similarly high RNA expression of the genes. Both EC and iPSC, but not ESC, can differentiate into TER. A study showed that xenograft with iPSC could develop into TER or into EC depending on the expression level of genes for pluripotency [36]. Therefore, the data favors the importance of the biologic similarity between EC and iPSC.

Knockdown studies support that our candidate genes are important. Knockdown of *PRAME* showed that PRAME inhibited that SE differentiated into TER [37]. Knockdown of *LIN28A* showed that LIN28A stimulates expression of *LIN28A, NANOG*, and *POU5F1* [38].

Nettersheim argued that TGCT developed based on a bidirectional plasticity [9,10]. The plasticity implies that SE develops into EC, as well as EC develops into SE. However, GCNIS progresses into TGCT [2] and EC progresses into the differentiated histologic types of NSGCT as indicated by Dixon and Moore [4]. Therefore, the histologic types of TGCT are linked by a unidirectional progression.

The Genomic Cancer Atlas (TGCA) extensively reported RNA findings in relation to the development of TGCT [12]. TGCA supported that the histologic types of TGCT develop in two lines for the development. However, our analysis of 24 candidate genes in the TGCA RNAseq dataset supports that TCGT develops in one-line progression among the histologic types.

The Vogelstein group indicates that a total of three mutations of oncogenes and tumor suppressor genes are sufficient to drive the malignant transformation [39], and that epigenetic regulations can substitute for the mutations in the malignant transformation. The iPSC shows that a panel of four genes of pluripotency can transform somatic cells into iPSC [32,33]. Therefore, four genes for pluripotency can reprogram NT into EC at least in hypotetraploid germ cells.

In TGCT, NSGCT often includes a combination of the histologic types [40]. However, a multivariate analysis showed that the various combinations of histologic types had no prognostic implications.

Future studies could further investigate how epigenetics regulates our candidate genes. It would be interesting to analyze whether RNA expression of the candidate genes among the histologic types of TGCT are associated with methylations and acetylations of lysines on histone 3 of promoters and enhancers for the candidate genes.

Our meta-analysis has limitations. It is not a systematic review. Pathologists accept that genes for pluripotency are important in elder patients for the development of GCT [41,42,43,44]. But most pathologists are not convinced that young adult patients, in general, develop TGCT due to a combined impact from a panel of genes for pluripotency.

In conclusion, *PRAME, KIT, SOX17, CCND2, KLF4, NANOG*, *POU5F1, DNMT3B, RB1*, and *LIN28A* are important in the pathogenesis of TGCT.

## 4. Materials and Methods

### 4.1. Data Sources

The meta-analysis evaluated two microarray datasets and an RNA sequencing (seq) dataset. The first microarray [13] used Affymetrix HG-U95A v2 (Thermo Fisher Scientific, Waltman, MA, USA) to analyze RNA expression. The microarray was available in Gene Expression Omnibus (GEO) at ascension number GSE 8607 (http:www.ncbi.gov.gen/query/ace.cgi?acc.GSE 8607). The first microarray included three samples of NT and 40 samples of SE.

The second microarray [14] used Agilent Human 1 A oligo assay (Agilent Technologies Inc, Palo Alto, CA, USA) to analyze RNA expression and was available in GEO at ascension number GSE 1818 (http:ww.ncbi.gov.gen/query/ace.cgi?acc.GSE 1818) and included three samples of NT, three samples of GCNIS, three samples of SE, five samples of EC, four samples of YST, four samples of YST, and one sample of CC.

The microarray summarized gene expression on log_2_ scale relative to a reference standard.

The TCGA Research Network made the RNAseq database [12] available at (http://portal.gdc.cancer.gov/legacy-archieve/).

The RNAseq dataset included 72 samples of SE, 27 samples of EC, 13 samples of YST, and 16 samples of TER.

Regarding the second microarray, we carried out inverse logarithmic transformation before we analyzed and reported the RNA expressions.

The original studies of the three datasets followed the Declaration of Helsinki.

### 4.2. Candidate Genes

The meta-analysis selected genes that previous publications had indicated were significant for the development of TGCT. Overall, our meta-analysis included six genes for pluripotency, five proto-oncogenes, four tumor suppressor genes, five genes for the three serum tumor markers, and four genes for biomarkers and a testis cancer antigen.

The six genes for pluripotency, *KLF4, LIN28, MYC, NANOG, POU5F1*, and *SOX17* were among the genes with the highest gene expression in GCNIS [22]. Of these genes, SE expresses SOX17 but not SOX2, whereas EC expresses SOX2 and not SOX17. Unfortunately, the two microarrays did not include SOX2, therefore, we were unable to undertake a meta-analysis of *SOX2*.

TGCT can include upregulated proto-oncogenes such as *CCND2, MDM2*, *MYC, NMYC*, *PTTG1*, and *CCNE1* [45,46,47,48]. TGCT can include downregulated tumor suppressor genes such as *CDK1N1A, CDKN2C, PTEN*, and *RB1* [49,50,51,52,53,54,55,56,57]. DNMT3B as a biomarker of TGCT.

Our list of candidate genes included *AFP, CGB5*, *LDHA, LDHB*, and *LDHC* for the genes for the serum tumor markers, alpha fetoprotein (AFP), human chorionic gonadotropin (hCG), and lactate dehydrogenase (EC 1.1.1.27, LDH), because the tumor markers are important for the treatment of TGCT. *AFP* and *CGB5* were secondary to the histologic types, and therefore the present meta-analysis included only limited analysis of the tumor marker genes.

### 4.3. Definitions and Evaluations

The meta-analysis defined *KLF4, LIN28A, NANOG*, and *POU5F1* as genes for pluripotency. We took a low combined *p* value from more than one dataset to indicate a role of the gene for the development of TGCT. We defined a gene as a driver gene if RNA expression of the gene increased stepwise from NT to SE/EC. We defined other significant genes as passenger genes.

We defined RNA expression of a gene as insignificant if the RNA expression of the gene did not differ statistically significantly among NT and the histologic types of TGCT.

A high RNA expression in two or more histologic types indicated an overlap of gene expression as a link as a histologic type progresses to the next. A significant change in RNA expression among histologic types indicated that the gene contributed to the development among the histologic types. We defined a significantly high RNA expression in only one histologic type as a genetic signature.

### 4.4. Statistical Analyses

The meta-analysis gave priority to comparisons among gene expressions in histologic types of TGCT that could be based on more than one dataset. We showed the combined *p* values based on the *p* values for comparisons of RNA expression of histologic types of the individual datasets using the meta-analytic method of the Fisher’s test [15].

The meta-analysis did not substitute missing data in the datasets of RNA expression in a tissue sample. The RNA expressions of the candidate genes had non-parametric distributions. Therefore, the meta-analysis used the median values for the RNA expressions of the genes and the Kruskal–Wallis test as we compared two groups of histologic types. All statistical tests were two sided. We considered a *p* value < 0.05 as statistically significant.

We carried out the statistical analyses with Stata 14.2 (Stata Corp., TX, USA).

## Figures and Tables

**Table 1:** RNA expressions of genes with overlaps among histologic types.

Histologies	Genes	No Patients	Combined *p* Values
SvEYT	*PRAME*		6 × 10^−5^
	*KLF4*		1.4 × 10^−5^
EvYT	*CCND2*	160	8.7 ×10^−5^
	*POU5F1*	160	1 × 10^−5^
	*RB1*	160	1 × 10^−5^
	*DNMT3B*	160	5.2 × 10^−5^

Abbreviations: SvEYT, SE versus EC, YST, and TER; EvYT, EC versus YST and TER.

**Table 2:** RNA expressions of genes that separate the histologic types.

Histologies	Genes	No. of Patients	Combined *p* Values
NvS	*KLF4*	60	0.042
	*KIT*	60	0.0022
	*RB1*	60	0.008
	*POUF1*	60	0.0019
	*CCND2*	60	0.0011
SvE	*SOX17*	160	3.4 × 10^−5^
	*KIT*	160	3.4 ×10^−5^
	*POU5F1*	160	0.0019
	*LIN28A*	160	2.1 × 10^−4^
	*DNMT3B*	160	3.5 × 10^−5^

Abbreviations: NvS, NT versus SE; SvE, SE versus EC.
